# A genomic screen for long noncoding RNA genes epigenetically silenced by aberrant DNA methylation in colorectal cancer

**DOI:** 10.1038/srep26699

**Published:** 2016-05-24

**Authors:** Kohei Kumegawa, Reo Maruyama, Eiichiro Yamamoto, Masami Ashida, Hiroshi Kitajima, Akihiro Tsuyada, Takeshi Niinuma, Masahiro Kai, Hiro-o Yamano, Tamotsu Sugai, Takashi Tokino, Yasuhisa Shinomura, Kohzoh Imai, Hiromu Suzuki

**Affiliations:** 1 Department of Molecular Biology, Sapporo Medical University, Sapporo, Japan; 2 PRESTO, Japan Science and Technology Agency, Kawaguchi, Saitama, Japan; 3 Department of Gastroenterology, Rheumatology and Clinical Immunology, Sapporo Medical University, Sapporo, Japan; 4 Department of Gastroenterology, Akita Red Cross Hospital, Akita, Japan; 5 Department of Diagnostic Pathology, Iwate Medical University, Iwate, Japan; 6 Medical Genome Science, Research Institute for Frontier Medicine, Sapporo Medical University School of Medicine, Sapporo, Japan; 7 Center for Medical Innovation, The Institute of Medical Science, The University of Tokyo, Japan.

## Abstract

Long noncoding RNAs (lncRNAs) have emerged as key components in multiple cellular processes, although their physiological and pathological functions are not fully understood. To identify cancer-related lncRNAs, we screened for those that are epigenetically silenced in colorectal cancer (CRC). Through a genome-wide analysis of histone modifications in CRC cells, we found that the transcription start sites (TSSs) of 1,027 lncRNA genes acquired trimethylation of histone H3 lysine 4 (H3K4me3) after DNA demethylation. Integrative analysis of chromatin signatures and the DNA methylome revealed that the promoter CpG islands (CGIs) of 66 lncRNA genes contained cancer-specific methylation. By validating the expression and methylation of lncRNA genes in CRC cells, we ultimately identified 20 lncRNAs, including ZNF582-AS1, as targets of epigenetic silencing in CRC. *ZNF582-AS1* is frequently methylated in CRC cell lines (87.5%), primary CRCs (77.2%), colorectal adenomas (44.7%) and advanced adenomas (87.8%), suggesting that this methylation is an early event during colorectal tumorigenesis. Methylation of *ZNF582-AS1* is associated with poor survival of CRC patients, and ectopic expression of ZNF582-AS1 suppressed colony formation by CRC cells. Our findings offer insight into the association between epigenetic alterations and lncRNA dysregulation in cancer and suggest that ZNF582-AS1 may be a novel tumor-suppressive lncRNA.

It was long believed that RNA functions solely to carry information encoded in genes to proteins; however, recent work has revealed the biological significance of noncoding RNAs. In addition to well-known short noncoding RNAs, such as microRNAs (miRNAs), long noncoding RNAs (lncRNAs) have emerged as key players in a wide variety of cellular processes, including chromatin modification, genome imprinting, X-chromosome inactivation and regulation of miRNA function^1^,^2^,^3^. lncRNAs share many of the biological characteristics of mRNAs. For instance, lncRNAs range from 200 nucleotides to more than 10 kb in length, and they are spliced and polyadenylated; however, lncRNAs do not contain open reading frames (ORFs). Moreover, recent advances in the ribosome profiling demonstrated that there is a clear gap between protein-coding transcripts and noncoding transcripts with respect to ribosomal occupancy, which indicates that lncRNAs do not encode proteins^4^.

In recent years, several studies have implicated lncRNAs in various malignancies. For instance, HOTAIR has been reported as being overexpressed in breast cancer, colorectal cancer (CRC) and gastrointestinal stromal tumors^5^,^6^,^7^. Through direct interaction with histone modification complexes, HOTAIR suppresses its target genes, and overexpression of HOTAIR is strongly associated with cancer metastasis and poor prognosis^5^. By contrast, growth arrest-specific 5 (GAS5) is a tumor-suppressor lncRNA that sensitizes cells to apoptosis by acting as a glucocorticoid receptor antagonist^8^. GAS5 overexpression suppresses cancer proliferation and induces apoptosis in breast cancer cell lines, and its expression is downregulated in human breast tumors compared with the normal breast epithelium^9^. More recently, CCAT2 was identified as a novel lncRNA overexpressed in CRC, in which it promotes metastasis and chromosomal instability^10^. These results suggest that lncRNAs can act as oncogenes or tumor suppressors, although the functions of the majority of lncRNAs remain to be determined.

Epigenetic gene silencing in association with promoter CpG island (CGI) hypermethylation is one of the common mechanisms by which tumor suppressor genes are inactivated during tumorigenesis^11^. In addition to DNA methylation, histone modification is also tightly associated with chromatin structure and gene transcription. For instance, trimethylation of histone H3 lysine 4 (H3K4me3) and lysine 36 (H3K36me3) are active transcription marks. H3K4me3 is enriched at the transcription start sites of actively transcribed genes, whereas H3K36me3 is enriched along the length of the transcribed regions. Guttman and colleagues have shown the usefulness of such chromatic signatures for the identification of genomic regions from which large intergenic noncoding RNAs (lincRNAs) are transcribed in mammals^12^.

In cancer cells, promoter CGI hypermethylation is associated with a loss of H3K4me3 and concomitant enrichment of repressive histone modifications, such as H3K9me3 and H3K27me3. We have previously demonstrated that chromatin signatures are powerful tools for the identification of miRNA genes epigenetically silenced in CRC^13^. In addition, we and others have shown that a number of miRNA genes are targets of epigenetic silencing in various types of cancer^14^,^15^,^16^,^17^. This information led us to speculate that cancer-related lncRNA genes may also be targets of epigenetic dysregulation in cancer. In the present study, we aimed to identify cancer-related lncRNAs through integrative genome-wide analysis of histone modification and DNA methylation in CRC cells. We also show that chromatin signatures before and after the removal of DNA methylation led to the robust identification of lncRNA genes that are epigenetically regulated in CRC.

## Results

### Epigenomic profiles of lncRNA genes in CRC

**Figure 1:**
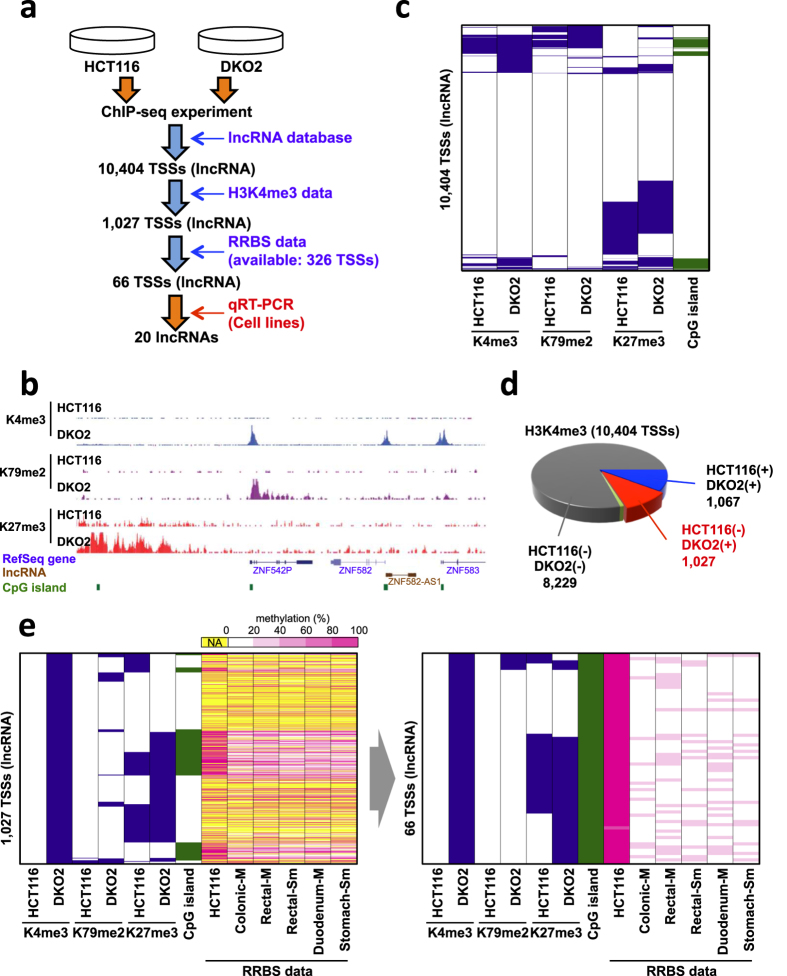
Screen for epigenetically silenced lncRNA genes in CRC. (**a**) Workflow of the screen to identify lncRNAs silenced in association with aberrant CGI methylation in CRC. (**b**) Representative results of a ChIP-seq analysis in HCT116 and DKO2 cells. (**c**) Heat map showing the presence (blue) or absence (white) of histone modifications (H4K4me3, H3K79me2 and H3K27me3) at the TSS regions of lncRNA genes in HCT116 and DKO2 cells. The presence (green) or absence (white) of a CGI is also indicated on the right. (**d**) The fraction of TSSs with an H3K4me3 mark in HCT116 and DKO2 cells. Shown are the numbers of TSSs with the indicated H3K4me3 status in the two cell lines. (**e**) Heat maps showing the histone modifications at selected TSSs in CRC cells. Shown is the DNA methylation status obtained from RRBS data sets for HCT116 and normal gastrointestinal tissues. A set of 1,027 TSSs with increased H3K4me3 in DKO2 cells is shown on the left, and 66 TSSs with cancer-specific CGI methylation are shown on the right. Colonic-M, colonic mucosa; Rectal-M, rectal mucosa; Rectal-Sm, rectal smooth muscle; Duodenum-M, duodenum mucosa; Stomach-Sm, stomach smooth muscle; NA, not available.

In order to identify epigenetically dysregulated lncRNA genes in CRC, we initially examined the chromatin signatures of lncRNA genes in CRC cells. Utilizing the ChIP-seq data sets from our previous study (Fig. 1a)^13^, we analyzed the H3K4me3, H3K27me3 and H3K79me2 status throughout the genomes of the HCT116 CRC cell line and isogenic *DNMT1*/*DNMT3B* double-knockout cells (DKO2), in which global DNA methylation is almost completely eliminated^18^ (Fig. 1b, Supplementary Figure S1). By customizing the published lncRNA database^19^, we obtained an annotation database covering a total of 10,404 transcription start sites (TSSs) for lncRNA genes. We then assessed the histone modification status in the TSS regions of the lncRNA genes in HCT116 and DKO2 cells. Among the 10,404 TSS regions, only 2,175 (20.9%) were positive for H3K4me3 marks in HCT116 and/or DKO2 cells, suggesting that a relatively limited number of lncRNA genes are transcribed in these cells (Fig. 1c,d). We then compared the H3K4me3 status in HCT116 and DKO2 cells and searched for regions that acquire an H3K4me3 mark after DNA demethylation. A total of 1,027 TSS regions were found to be associated with upregulated H3K4me3 in DKO2 cells, which is indicative of transcriptional activation after DNA demethylation (Fig. 1d). Of those 1,027 regions, 399 (38.9%) also had an H3K27me3 mark in HCT116 cells, which was often upregulated in DKO2 cells (Fig. 1b,e, Supplementary Figure S1). By contrast, differences in H3K79me2 marks between the two cell lines were substantially smaller (Fig. 1c,e, Supplementary Figure S1).

To identify lncRNA genes showing CGI methylation, we next combined the chromatin signatures with DNA methylome data sets. Using reduced representation bisulfite sequencing (RRBS) data sets for HCT116 cells and normal gastrointestinal tract mucosal tissue from the colon, rectum, duodenum and stomach, we extracted the DNA methylation data for the TSS regions of lncRNA genes, and stratified the genes into five categories based to their methylation levels: 0–20%, 20–40%, 40–60%, 60–80% and 80–100%. We then searched for genes that exhibited high levels of methylation (>60%) in HCT116 cells and low levels (<40%) in normal gastrointestinal tissues. Of the 1,027 genes with upregulated H3K4me3 in DKO2 cells, RRBS data were available for 326, and 74 satisfied the search criteria. Among them, 66 genes had CGIs around their TSS regions, and we selected those genes for further analysis (Fig. 1e, Supplementary Table S1). In addition, to evaluate the reliability of our strategy to identify epigenetically silenced genes with cancer-specific DNA methylation, we performed the same analysis for the protein-coding genes using the same epigenome datasets (Supplementary Figure S2). The list of genes identified by our analysis included a number of genes previously reported to be methylated in CRC, which confirmed the robustness of our screening method (Supplementary Table S2).

### Validation of epigenetically silenced lncRNA genes in CRC

**Figure 2:**
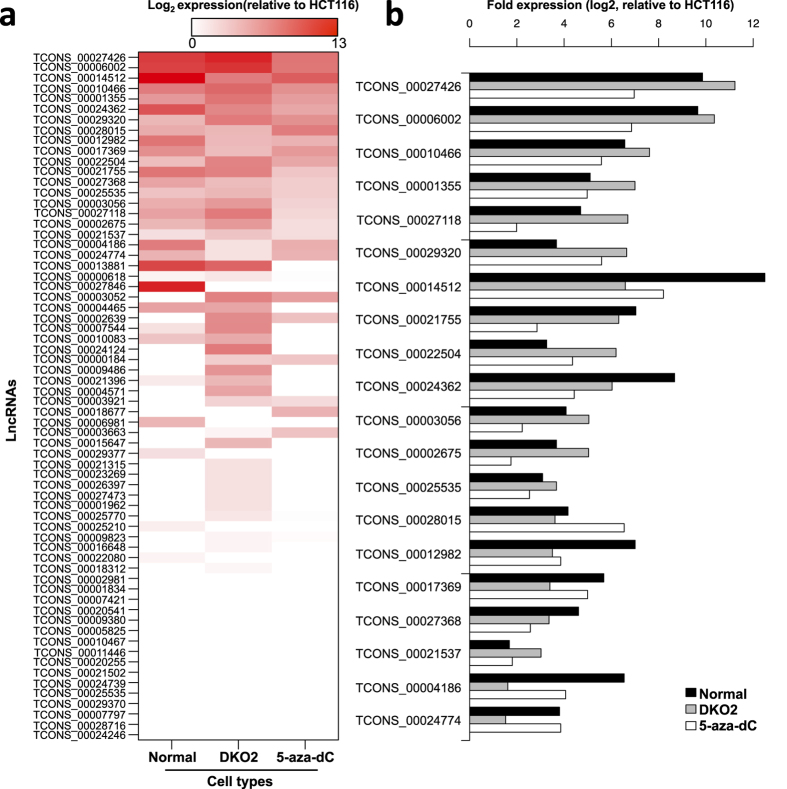
Analysis of lncRNA expression. (**a**) Heat map showing the expression of 66 lncRNAs in normal colon, HCT116 cells treated with 5-aza-dC and DKO2 cells. Expression levels of the indicated lncRNAs were determined by quantitative RT-PCR, after which, the results were normalized to the expression levels in untreated HCT116 cells. The color scale indicates the relative expression levels on a log2 scale. (**b**) Expression of 20 lncRNAs in normal colon, HCT116 cells treated with 5-aza-dC and DKO2 cells. The results were normalized to the expression levels in untreated HCT116 cells.

We next performed quantitative reverse transcription-PCR (RT-PCR) to assess the expression levels of the identified lncRNAs in HCT116 cells with or without 5-aza-2’-deoxycytidine (5-aza-dC) treatment and in DKO2 cells and a sample of normal colonic mucosa (Fig. 2a). We searched for lncRNAs that (a) were expressed in normal colon and downregulated in HCT116 cells and (b) were upregulated by pharmacological (5-aza-dC) or genetic depletion of DNMTs (DKO2 cells). Of the 66 candidates described above, 31 were expressed in the normal colon, and 28 were downregulated (>2-fold) in HCT116 cells compared with the normal colon. In addition, 26 lncRNAs were upregulated (>2-fold) by 5-aza-dC treatment in HCT116 cells, and 44 were upregulated in DKO2 cells compared with their parental HCT116 cells. Taken together, our findings indicate that 20 lncRNAs fully satisfied the two criteria used for this search (Fig. 2b).

**Figure 3:**
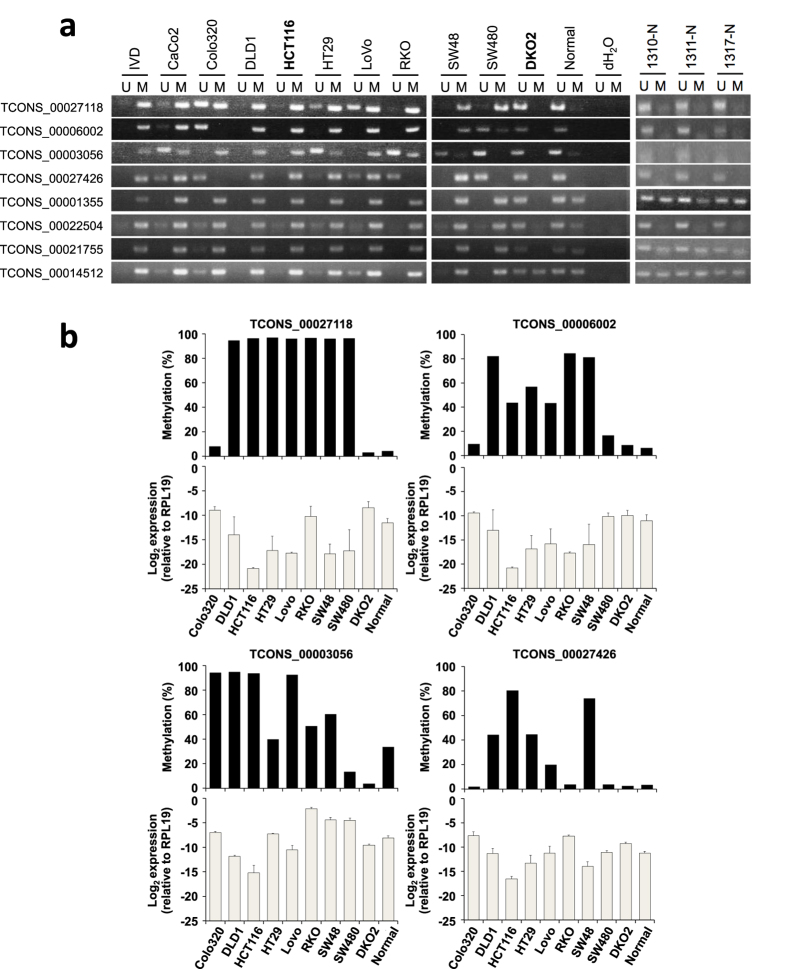
DNA methylation and expression of lncRNA genes in CRC cells. (**a**) Methylation-specific PCR analysis of the promoter CGIs of the indicated lncRNA genes in CRC cell lines, a normal colonic tissue from a healthy individual (24 yo), and normal colonic tissues from CRC patients (1310-N, 74 yo; 1311-N, 75 yo; 1317-N, 79 yo). Bands in the “M” lanes are PCR products obtained with methylation-specific primers. Those in the “U” lanes are products obtained with unmethylation-specific primers. *In vitro*-methylated DNA (IVD) serves as a positive control. (**b**) The relationship between DNA methylation and expression of lncRNA genes in CRC cells and a normal colonic tissue. Shown are the results of bisulfite pyrosequencing (black bars) and quantitative RT-PCR (white bar) analysis of the four selected lncRNA genes. RT-PCR results were normalized to the internal RPL19 expression.

To assess the DNA methylation status of the identified lncRNA genes, we next performed methylation-specific PCR (MSP) in a series of CRC cell lines and in normal colonic mucosa from a healthy individual. In addition, we also analyzed normal colon mucosa from elderly CRC patients (age, 74 yo, 75 yo and 79 yo), in order to exclude age-related methylation. MSP analysis revealed that the CGIs in lncRNA genes were methylated at various frequencies in the tested cell lines (Fig. 3a, Supplementary Figure S3). We selected four genes that were frequently methylated in CRC cell lines (*TCONS_00027118, TCONS_00006002, TCONS_00003056* and *TCONS_00027426*) for further analysis. To perform quantitative DNA methylation analysis, we carried out bisulfite pyrosequencing and found these genes were methylated at high levels in CRC cells (Fig. 3b). Quantitative RT-PCR showed that there were significant negative correlations between the methylation and expression levels of three genes (*TOCNS_00027118*, R = −0.75, p = 0.01; *TCONS_00006002*, R = −0.65, p = 0.04; *TCONS_00027426*, R = −0.87, p = 0.001) (Fig. 3b). We also noted that three of the genes (*TCONS_0027118, TCONS_00006002* and *TCONS_00027426*) showed elevated methylation levels in multiple CRC cell lines, whereas the methylation levels were minimal in normal colonic tissue (Fig. 3b, Supplementary Figure S3).

**Figure 4:**
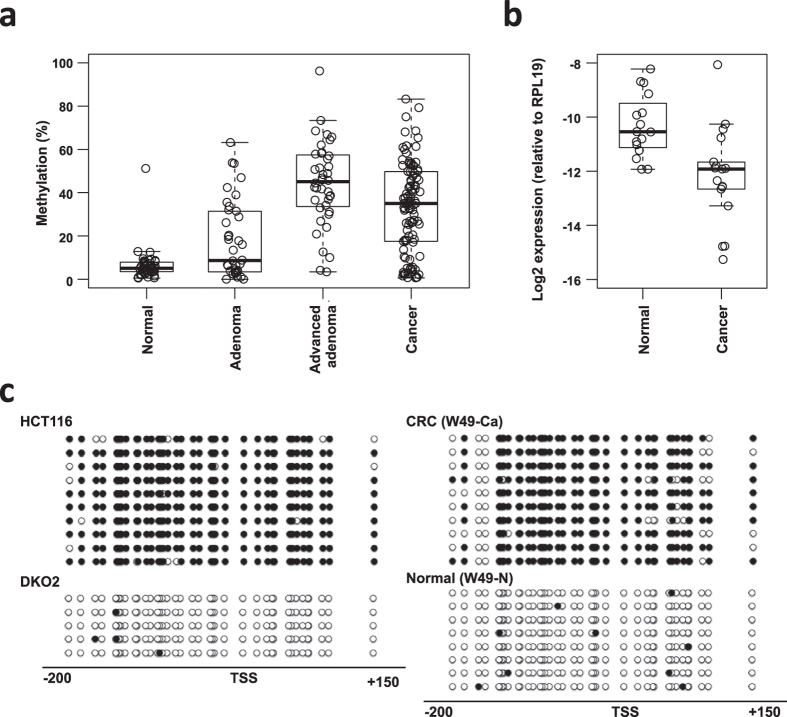
DNA methylation and expression of *TCONS_00027118* (*ZNF582-AS1*) in clinical samples. (**a**) Summary of bisulfite pyrosequencing of the CGI of *ZNF582-AS1* in normal colon (n = 46), colorectal adenomas (n = 38), advanced adenomas (n = 40) and primary CRCs (n = 101). Each dot represents a single specimen, and the first, second and third quartiles are shown as box plots. (**b**) Quantitative RT-PCR results for ZNF582-AS1 in normal colonic tissues (n = 16) and primary CRCs (n = 17). (**c**) Bisulfite sequencing results for the *ZNF582-AS1* CGI in HCT116, DKO2 and a pair of primary CRC (W49-Ca) and normal colon tissues (W49-N, age 84 yo). Open and filled circles represent unmethylated and methylated CpG sites, respectively.

We next performed bisulfite pyrosequencing to assess the methylation status of *TCONS_0027118, TCONS_00006002* and *TCONS_00027426* in a series of clinical samples (Fig. 4a, Supplementary Figure S4). We found that levels of *TCONS_00027118* (also known as *ZNF582-AS1*) methylation were frequently elevated (>15.0%) in primary CRC tissues (78/101, 77.2%) (Fig. 4a). Total RNA was available from 16 normal and 17 CRC tissues, and quantitative RT-PCR analysis revealed downregulated expression of ZNF582-AS1 in primary CRC tissues (Fig. 4b). *ZNF582-AS1* methylation was also elevated in colorectal adenomas (17/38, 44.7%) and advanced adenomas (36/40, 87.8%), indicating that methylation of *ZNF582-AS1* is an early event during colorectal tumorigenesis (Fig. 4a). Bisulfite sequencing analysis confirmed dense methylation of *ZNF582-AS1* in HCT116 cells whereas almost completely unmethylated in DKO2 cells (Fig. 4c). Results of the bisulfite pyrosequencing in Fig. 4a were further validated by analyzing a pair of primary CRC and a normal colon tissue from a CRC patient (age, 84 yo) (Fig. 4c). Methylation of *TCONS_00006002* and *TCONS_00027426* was also frequently observed in primary CRCs (39.1% and 20.0%) and advanced adenomas (52.6% and 23.1%) (Supplementary Figure S4), suggesting that multiple lncRNA genes are potential targets of epigenetic silencing in CRC.

### Methylation and functional analysis of *ZNF582-AS1* in cancer

**Figure 5:**
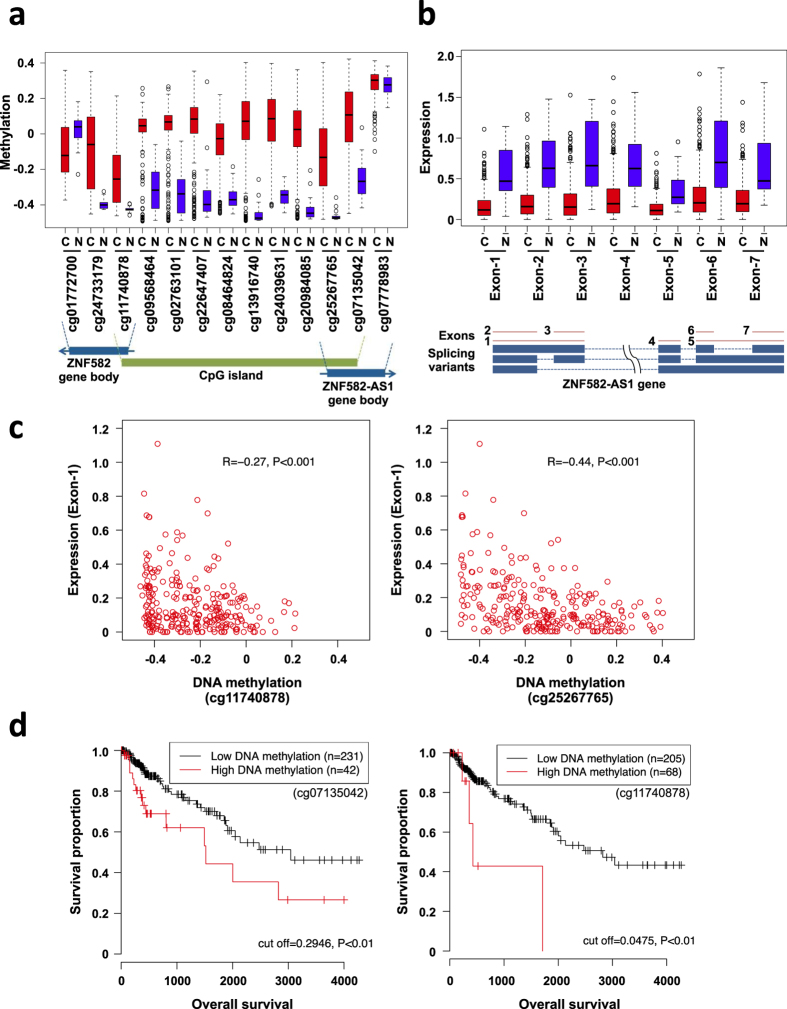
DNA methylation and ZNF582-AS1 expression analyzed in primary CRCs using TCGA data sets. (**a**) Levels of DNA methylation in the promoter CGI and gene bodies of *ZNF582* and *ZNF582-AS1* in normal colon (N; n = 38) and primary CRCs (C; n = 285). β-values for the Infinium HumanMethylation450 BeadChip at respective CpG sites are shown as box plots. Probe IDs for the Infinium platform are indicated below. (**b**) Expression levels of the indicated ZNF582-AS1 exons in normal colon (n = 50) and primary CRCs (n = 364) determined by RNA-seq. Shown below are the gene structures of the transcriptional ZNF582-AS1 variants and regions analyzed by RNA-seq. (**c**) Correlations between DNA methylation at the indicated probe sets and expression of ZNF582-AS1 exon 1. The Pearson correlation coefficients and P values are shown. (**d**) Kaplan-Meier curves showing the effect of DNA methylation at the indicated probe sets on overall survival among CRC patients. The β-value cut-off for the respective probe sets, and the P values are also shown.

To further confirm the results summarized above in a large set of clinical samples, we used Infinium HumanMethylation450 BeadChip data and RNA-seq data obtained from primary CRC tissues in The Human Cancer Genome Atlas (TCGA) network study. As shown in Fig. 5a, *ZNF582-AS1* shares its promoter CGI with a protein-coding gene, *ZNF582*, which is transcribed in the opposite direction. In the normal colon, methylation levels were low throughout the entire CGI region of *ZNF582*/*ZNF582-AS1*, whereas they were significantly elevated in CRC (Fig. 5a). By contrast, CpG sites within the gene bodies of both *ZNF582* and *ZNF582-AS1* were similarly methylated in CRC and normal colonic tissue. RNA-seq data revealed that all *ZNF582-AS1* exons were transcribed in normal colonic tissue, whereas the expression levels were significantly downregulated in CRCs (Fig. 5b). We observed a similar expression pattern for ZNF582 in CRC and normal colonic tissue, indicating that the CGI region functions as a bidirectional promoter for these genes (Supplementary Figure S5). We also observed inverse relationships between the methylation levels at multiple probe sets and the levels of ZNF-582-AS1 expression in cancer tissues (methylation of cg11740878 and expression of exon 1, R = −0.27, p < 0.001; cg25267765 and exon 1, R = −0.44, p < 0.001; Fig. 5c, other probes are shown in Supplemental Figure S5). Notably, higher levels of methylation at multiple CpG sites in the CGI of *ZNF582-AS1* were associated with poorer overall survival in CRC patients (Fig. 5d).

**Figure 6:**
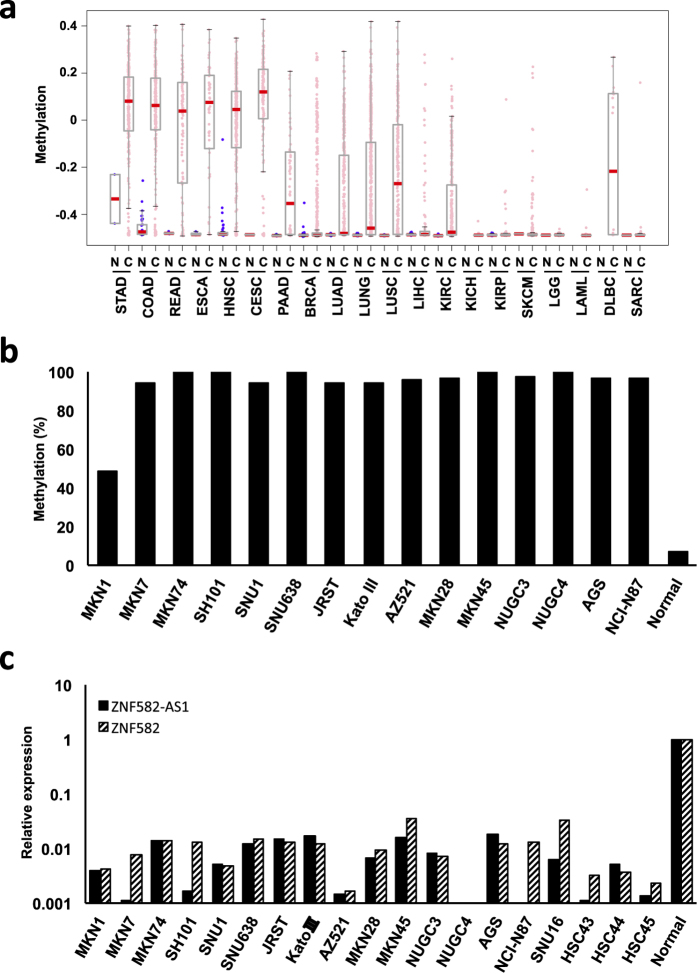
DNA methylation and ZNF582-AS1 expression in cancers of various origins. (**a**) Levels of DNA methylation in the promoter CGI of *ZNF582-AS1* in the indicated tumors (C) and corresponding normal tissues (N) analyzed using TCGA data sets. STAD: stomach adenocarcinoma, COAD: colon adenocarcinoma, READ: rectum adenocarcinoma, ESCA: esophageal carcinoma, HNSC: head and neck squamous cell carcinoma, CESC: cervical and endocervical cancer, PAAD: pancreatic adenocarcinoma, BRCA: breast invasive carcinoma, LUAD: lung adenocarcinoma, LUNG: lung cancer, LUSC: lung squamous cell carcinoma, LIHC: liver hepatocellular carcinoma, KIRC: kidney clear cell carcinoma, KICH: kidney chromophobe, KIRP: kidney papillary cell carcinoma, SKCM: skin cutaneous melanoma, LGG: brain lower grade glioma, LAML: acute myeloid leukemia, DLBC: diffuse large B-cell lymphoma, SARC: sarcoma. (**b**) Levels of *ZNF582-AS1* methylation in gastric cancer cell lines and normal gastric mucosa analyzed using bisulfite pyrosequencing. (**c**) Quantitative RT-PCR analysis of ZNF582-AS1 and ZNF582 in a series of gastric cancer cell lines and normal stomach tissues. The results are normalized to internal RPL19 expression.

We also determined whether *ZNF582-AS1* methylation is a common event in human malignancies. Using the Infinium BeadChip data sets from TCGA studies, we assessed *ZNF582-AS1* methylation in various cancers. We found that levels of *ZNF582-AS1* methylation were elevated in gastric, esophageal, head and neck, cervical and pancreatic cancers as well as in lung squamous cell carcinomas, whereas the methylation levels were low in normal tissues from the corresponding organs (Fig. 6a). We also found that the promoter CGI of *ZNF582-AS1* was methylated in a majority of the gastric cancer cell lines tested (Fig. 6b). Consistent with its methylation status, expression of both ZNF582-AS1 and ZNF582 was downregulated in the gastric cancer cell lines compared with normal stomach mucosal tissue (Fig. 6c). It thus appears that *ZNF582-AS1* methylation is a common event in human cancers, and its silencing may be causally related to tumorigenesis in multiple organs.

**Figure 7:**
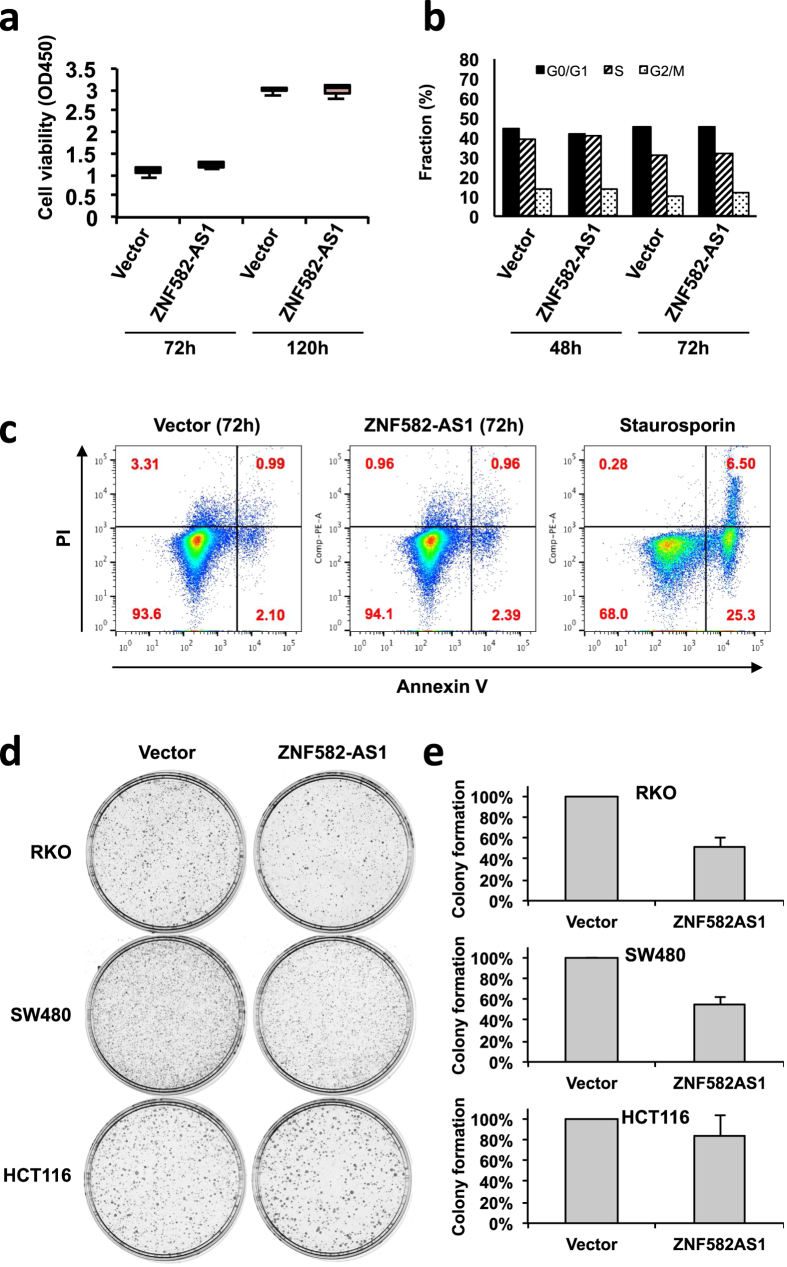
Functional analysis of ZNF582-AS1 in CRC cells. (**a**) RKO cells were transfected with expression constructs encoding ZNF582-AS1 or a control vector, after which, cell viability was assessed in WST-8 assays at the indicated time points. (**b**) Cell cycle distribution of RKO cells transfected with ZNF582-AS1 vector or a control vector was measured by pulse EdU incorporation and DNA content in flow cytometric analysis. Percentages of cells in the G0/G1, S, and G2/M phases are shown. (**c**) RKO cells transfected with ZNF582-AS1 or a control vector were incubated with Annexin V and PI, and labeled cells were analyzed on flow cytometry. Annexin V-positive cells indicate the apoptotic population, and double-positive cells indicate the necrotic population. Cells treated with Staurosporin serve as a positive control for the experiment. (**d**) Representative results of a colony-formation assay performed with the indicated CRC cells. (**e**) Relative colony formation efficiencies of CRC cells transfected with ZNF582-AS1 or a control vector. Shown are means of three replicates; the error bars represent standard deviations.

We next attempted to clarify the functional role of ZNF582-AS1 in CRC. To induce ectopic expression of ZNF582-AS1 in CRC cells, we transfected cells with a ZNF582-AS1 expression construct or an empty vector and confirmed the expression using quantitative RT-PCR (Supplementary Figure S6). We next examined whether ectopic ZNF582-AS1 expression could affect cancer cell viability. A WST-8 assay and flow cytometry analysis revealed that ZNF582-AS1 did not affect cell viability or proliferation, the cell cycle or apoptosis in CRC cell lines, HCT116, RKO and SW480 (Fig. 7a–c, Supplementary Figure S6 and data not shown). Because methylation of the CGI region is responsible for the transcriptional silencing of both ZNF582-AS1 and ZNF582, we speculated that restoration of ZNF582-AS1 alone may not be sufficient to exert its effect. To test this possibility, HCT116 cells were co-transfected with ZNF582-AS1 and ZNF582 expression vectors; however, the co-expression of the two genes also did not inhibit cellular proliferation, suggesting that the 2 genes may have independent functions (Supplementary Figure S6). To obtain further insight into the function of ZNF582-AS1, we performed gene expression microarray analysis using HCT116 cells transiently transfected with a control vector or a ZNF582-AS1 expression construct. Gene Ontology enrichment analysis and pathway analysis revealed that genes associated with G-protein-coupled receptor signaling pathway were significantly enriched among the 2,324 genes downregulated (>1.5-fold) by ectopic ZNF582-AS1 expression (Supplementary Figure S6). Finally, to evaluate the long-term effect of ZNF582-AS1, we performed colony-formation assays and found that ZNF582-AS1 suppressed colony formation in 2 out of 3 CRC cell lines (RKO and SW480), but it did not show a suppressive effect in HCT116 cells (Fig. 7d,e). These results suggest that ZNF582-AS1 may act as a tumor suppressor in CRC, but further studies are necessary to clarify its function.

## Discussion

Recent evidence suggests that DNA methylation is associated with the transcriptional dysregulation of lncRNA genes in cancer. For instance, maternally expressed gene 3 (*MEG3*) is reportedly downregulated in association with promoter hypermethylation in meningioma and ovarian and gastric cancers^20^,^21^,^22^, and a recent deep bisulfite sequencing analysis identified hypermethylation of the lncRNA gene colorectal adenocarcinoma hypermethylated (*CAHM*) in CRC^23^. By contrast, *APF1-AS1* was found to be hypomethylated and overexpressed in Barrett’s esophagus and esophageal adenocarcinoma^24^. Recently, Zhi *et al*. performed a comprehensive analysis of the patterns of lncRNA gene methylation by re-annotating the probes on the Infinium HumanMetylation450 BeadChip^25^. By comparing the DNA methylation array and RNA-seq results obtained with the H1-hESC cell line, they found an inverse association between hypermethylation in the promoter region and expression of lncRNA genes. The authors also analyzed lncRNA gene methylation in 20 different types of cancer and found that 2,461 lncRNAs could be subcategorized according to their methylation patterns.

In the present study, we provide a comprehensive view of the patterns of histone modification at lncRNA genes in CRC cells. As with protein-coding genes, lncRNA gene transcription is tightly associated with active and repressive histone marks. We were therefore able to utilize histone modifications to screen for epigenetically dysregulated lncRNAs in CRC cells. The H3K4me3 mark was found in only 20.9% (2,175 of 10,404) of the predicted TSS regions of lncRNA genes in HCT116 and/or DKO cells, suggesting that patterns of lncRNA expression are highly tissue specific^19^. Notably, the H3K4me3 mark was upregulated in approximately 10% (1,027 of 10,404) of lncRNA genes after DNA demethylation in HCT116 cells, suggesting that a substantial number of lncRNAs are potential targets of epigenetic silencing in cancer cells. We also found that the chromatin signatures in epigenetically silenced lncRNA genes showed a pattern similar to those in protein-coding and miRNA genes. For instance, earlier studies showed that epigenetically silenced genes retain repressive histone marks, even after DNA demethylation^13^,^26^,^27^. In addition, another study showed that DNA demethylation never restores H3K79me2 in tumor suppressor genes with CGI methylation in cancer cells^28^. One of the limitations of our study is that the restoration of the H3K4me3 marks may not indicate the transcriptional reactivation of the gene. We thus carefully confirmed the expression of the selected lncRNA genes in CRC cells with or without demethylation.

To further identify lncRNA genes with aberrant DNA methylation, we used RRBS data sets to compare the DNA methylation statuses of CRC cells and normal gastrointestinal tissue. Although RRBS covered only one-third of the predicted TSS regions in lncRNA genes, we were able to identify 66 lncRNAs that satisfied the criteria for cancer-specific methylation and ultimately identified 20 lncRNAs as targets of epigenetic silencing in CRC. Among the lncRNA genes identified, we found that *ZNF582-AS1* is frequently methylated in CRCs as well as in other cancers. *ZNF582-AS1* shares its promoter CGI with a protein-coding gene transcribed in the opposite direction, indicating bidirectional promoter function. Earlier studies have reported the methylation of *ZNF582* in cervical and oral cancers^29^,^30^, but this is the first report of CGI methylation associated with the silencing of both *ZNF582-AS1* and *ZNF582*. We previously reported a similar pattern for miRNA and protein-coding genes in CRC. A CGI located in the proximal upstream of *miR-34b/c* acts as a bidirectional promoter, and its methylation is associated with inactivation of both *miR-34b/c* and *BTG4*^14^.

Although the actions of most lncRNAs remain unknown, dozens of examples of biologically functional lncRNAs have been reported. For instance, one group of lncRNAs act as “guides” that bind to specific proteins and direct their transport to specific targets^31^. HOTAIR suppresses the *HOXD* locus by recruiting the PRC2 complex in normal adult fibroblasts^32^ and regulates several hundred target genes in cancer cells^5^. Another class of lncRNAs may possess domains that bind various effector molecules and acts as a “scaffold.” Two examples of lncRNAs that act as scaffolds are ANRIL, which interacts with components of PRC1 and PRC2^33^,^34^, and KCNQ1OT1, which binds both G9a and PRC2^35^. A third class of lncRNAs acts as “decoys” that bind to and sequester protein or RNA targets^36^. PTENP1, a pseudogene of the tumor suppressor PTEN, acts as a decoy by binding to miRNAs that normally target PTEN^37^. It is thus likely that ZNF582-AS1 functions through interactions with other proteins and/or RNA molecules. In the present study, we found that ectopic expression of ZNF582-AS1 did not induce cell cycle arrest or apoptosis in CRC cells, but it suppressed colony formation by the cancer cells. The results of gene expression microarray analysis suggested that ZNF582-AS1 may affect the G-protein coupled receptor signaling pathway. However, although ZNF582-AS1 is significantly downregulated in HCT116 cells, ectopic expression of ZNF582-AS1 did not show a growth suppressive effect in HCT116 cells. Because of such limitations of our study, it is still possible that the correlation between methylation of *ZNF582-AS1* and the poorer survival of CRC patients may not be due to its tumor suppressive function. Further studies are necessary to unravel the function and roles of ZNF582-AS1 in CRC development.

Our data also suggest that HCT116 cells may lack factors required for ZNF582-AS1 to exert its full function. Because the same promoter regulates the transcription of both *ZNF582-AS1* and *ZNF582*, we hypothesized that these genes may functionally interact with one another. However, the co-transfection of the two genes did not suppress HCT116 cell growth. A recent study of the cellular localization of lncRNAs found no specific correlation between the expression or localization patterns of lncRNA-mRNA transcription pairs^38^. This result suggests that the functions of ZNF582-AS1 and ZNF582 are distinct from one another, although further studies will be necessary to clarify their roles in cancer cells.

The high frequency of *ZNF582-AS1* methylation in CRC and advanced adenomas suggests it could be a biomarker for the early detection of CRCs. In addition, elevated levels of *ZNF582-AS1* methylation may be a predictive marker of outcome in CRC patients. In recent years, a number of studies have shown that detection of aberrant DNA methylation in bodily fluids (e.g., blood, urine and stool) is a promising strategy for noninvasive cancer diagnosis. In parallel with this study, we assessed the clinical utility of DNA methylation detected in bowel lavage fluid (BLF) for CRC screening^39^. We collected BLF specimens from individuals who were undergoing colonoscopy and tested a series of genes including *ZNF582-AS1* (previously called *LOC386758*). By comparison with the colonoscopy results, we found that a panel of three genes (*miR-124-3, LOC386758* and *SFRP1*) could be a useful biomarker for the detection of CRC. Given that elevated levels of *ZNF582-AS1* methylation have been observed in several human malignancies, further studies evaluating its clinical utility as a diagnostic biomarker appear warranted.

In summary, we provide evidence that lncRNA genes are frequent targets of epigenetic silencing in CRC. Similar to protein-coding and miRNA genes, DNA methylation and histone modification are deeply involved in the dysregulation of lncRNA genes in cancer cells. Our results also suggest that methylation of the lncRNA genes could serve as a useful tumor marker, and that restoration of the silenced lncRNAs could be an effective anticancer therapy.

## Materials and Methods

### Ethics Statement

Informed consent was obtained from all patients before collection of the specimens. Approval for this study was obtained from the Institutional Review Board of Akita Red Cross Hospital and Sapporo Medical University. All methods were carried out in accordance with the approved guidelines.

### Cell lines and tissue specimens

Colorectal cancer cell lines and HCT116 cells harboring genetic disruptions within the *DNMT1* and *DNMT3B* loci (DKO2 cells) have been described previously^13^. Cells were treated with 1 μM 5-aza-dC for 72 h, and the drug and medium were refreshed every 24 h. A total of 105 primary CRC specimens were obtained as described previously^40^. Samples of adjacent normal colorectal mucosa were also collected from 46 patients. A total of 41 advanced adenoma and 38 adenoma specimens were obtained through endoscopic biopsy as described^39^,^40^. Advanced adenomas were defined as adenomas 1 cm or greater in diameter and/or containing villous components and/or high-grade dysplasia. Total RNA was extracted using an RNeasy Mini kit (Qiagen) or TRIZOL reagent (Invitrogen). Genomic DNA was extracted using the standard phenol-chloroform procedure or a DNeasy Blood & Tissue kit (Qiagen). Total RNA and genomic DNA of normal colon and stomach tissues from healthy individuals were purchased from BioChain, Ambion and Clontech.

### Chromatin immunoprecipitation

Chromatin immunoprecipitation (ChIP) and deep sequencing were performed as described previously^13^.

### Data analysis

An annotation data set for human lncRNA genes was obtained from the Human Body Map of lincRNAs at the Broad Institute^19^. Sequence data obtained from ChIP-seq experiments were mapped to the human genome (UCSC hg19) using Bowtie software (http://bowtie-bio.sourceforge.net/index.shtml). We defined H3K4me3 peaks using MACS1.4 software (http://liulab.dfci.harvard.edu/MACS/)^41^ in the default setting. Genes were defined as H3K4me3-positive when H3K4me3 peaks overlapped with regions encompassing TSSs (−2 kb to +2 kb relative to TSSs). H3K27me3 and H3K79me2 peaks were identified using the SICER algorithm (http://home.gwu.edu/~wpeng/Software.htm)^42^, and genes were defined as H3K27me3-positve or H3K79me2-positive when H3K27me3 or H3K79me2 peaks overlapped with regions encompassing TSSs (−5 kb to +5 kb relative to TSSs). Reduced representation bisulfite sequencing (RRBS) data sets for normal gastrointestinal tissues (normal colonic mucosa, rectal mucosa, rectal smooth muscle, stomach smooth muscle and duodenum mucosa) were obtained from the Roadmap Epigenomics Project database (The NIH Roadmap Epigenomics Mapping Consortium, http://www.roadmapepigenomics.org/data/). The RRBS data set for HCT116 was obtained from the ENCODE project (http://hgdownload.cse.ucsc.edu/goldenPath/hg19/encodeDCC/wgEncodeHaibMethylRrbs/). Methylation values for the CpG sites located in the promoter regions (−500 bp to +500 bp relative to TSSs) were obtained from the RRBS data sets, and the genes were categorized into 5 groups (0–20%, 20–40%, 40–60%, 60–80%, 80–100%) according to the methylation levels of their promoter regions. Genome-wide DNA methylation and expression data in The Cancer Genome Atlas Network (TCGA) data sets were obtained from the Cancer Genomics Browser (https://genome-cancer.ucsc.edu). Level 3 data sets obtained through Infinium HumanMethylation450 BeadChip and RNA sequencing were analyzed using R software (http://www.R-project.org/). Genomic locations are based on UCSC hg19 produced by the International Human Genome Sequencing Consortium. We also obtained locations of CGIs, RefSeq genes and UCSC genes from the UCSC hg19 data sets. Survival analysis was performed using the Cutoff Finder website (http://molpath.charite.de/cutoff/).

### Quantitative reserve transcription-PCR

Single-stranded cDNA was prepared using SuperScript III reverse transcriptase (Life Technologies), after which the integrity of the cDNA was confirmed by amplifying beta-actin (ACTB) and ribosomal protein L19 (RPL19). Quantitative reverse transcription-PCR (RT-PCR) was performed using SYBR Select Master Mix (Applied Biosystems) and a 7500 Fast Real-Time PCR System (Applied Biosystems). Relative expression levels of target lncRNAs were determined using the endogenous housekeeping genes ACTB and RPL19 as internal controls. Primer information is provided in Supplementary Table S3.

### DNA methylation analysis

Genomic DNA (1 μg) was modified with sodium bisulfite using an EpiTect Bisulfite kit (Qiagen). Methylation-specific PCR, bisulfite sequencing and bisulfite pyrosequencing were conducted as described previously^13^. The pyrosequencing reaction was performed using a PSQ96 system with a PyroGold reagent Kit (Qiagen), and the results were analyzed using Q-CpG software (Qiagen). For bisulfite sequencing, amplified PCR products were cloned into pCR2.1-TOPO vector (Life Technologies), and 10 clones from each sample were sequenced using an ABI3130x automated sequencer (Life Technologies). Primer sequences and PCR product sizes are listed in Supplementary S3.

### Expression vectors

Full-length cDNA encoding ZNF582-AS1 was PCR amplified using cDNA derived from HEK293 cells as a template, after which, the fragment was cloned into the pcDNA3.1 (Invitrogen) and pEBMulti (Wako) vectors. pFN21A HaloTag CMV Flexi Vector harboring the ZNF582 ORF was purchased from Kazusa DNA Research Institute.

### Cell viability and colony-formation assays

Cells were transfected with 1 μg of ZNF582-AS1 or ZNF582 expression vector or an empty vector using Lipofectamine 3000 according to the manufacturer’s instructions. The viability of the transfectants was then analyzed in water-soluble tetrazolium salt (WST-8) assays using a Cell Counting Kit-8 (Dojindo). For colony-formation assays, the cells were plated on 60-mm culture dishes 24 h after transfection and selected for 10 to 14 days with 1.0 mg/ml (RKO cells) or 0.6 mg/ml (HCT116 and SW480 cells) G418. The colonies were stained with Giemsa.

### Flow cytometry analysis

Cells were transfected with 2.5 μg of a ZNF582-AS1 expression vector or an empty vector as described above. Forty-eight to 96 h after transfection, the cells were harvested and subjected to flow cytometry analyses using ApoScreen Annexin V Apoptosis Kit (Southern Biotech) or Click-iT Plus EdU Alexa Fluor 647 Flow Cytometry Assay Kit (Life Technologies) and BD FACSCanto II (BD Biosciences). Data analysis was performed using the FlowJo software (FlowJo, LLC).

### Gene expression microarray analysis

HCT116 cells were transfected with a ZNF582-AS1 expression vector or an empty vector as described above. Seventy-two hours after transfection, total RNA was extracted and labeled using a Quick Amp Labeling Kit One-Color (Agilent Technologies), after which, the synthesized cRNA was hybridized to the SurePrint G3 Human GE 8 × 60K V2 microarray (Agilent Technologies) according to the manufacturer’s instructions. The microarray data were analyzed using GeneSpring GX version 13 (Agilent Technologies).

### Statistical analyses

The mean DNA methylation levels among different tumor types or organs were compared using unpaired, two-tailed Student’s t tests. DNA methylation levels were correlated with expression levels by calculating the Pearson’s correlation coefficients. Values of p < 0.01 (two-sided) were considered significant. Statistical analyses were performed using R software.

## Additional Information

**How to cite this article**: Kumegawa, K. *et al*. A genomic screen for long noncoding RNA genes epigenetically silenced by aberrant DNA methylation in colorectal cancer. *Sci. Rep.* **6**, 26699; doi: 10.1038/srep26699 (2016).

## Supplementary Material

Supplementary Information
