# Supplementary material for: A genomic screen for long noncoding RNA genes epigenetically silenced by aberrant DNA methylation in colorectal cancer
Source: Sci Rep. 2016 May 24;6:26699. doi: 10.1038/srep26699 (PMC4877637; doi:10.1038/srep26699)
Supplement: Supplementary Information [file srep26699-s1.pdf]

A genomic screen for long noncoding RNA genes epigenetically silenced by aberrant DNA methylation in colorectal cancer

Kohei Kumegawa, Reo Maruyama, Eiichiro Yamamoto, Masami Ashida, Hiroshi Kitajima Akihiro Tsuyada, Takeshi Niinuma, Masahiro Kai, Hiro-o Yamano, Tamotsu Sugai, Takashi Tokino, Yasuhisa Shinomura, Kohzoh Imai and Hiromu Suzuki

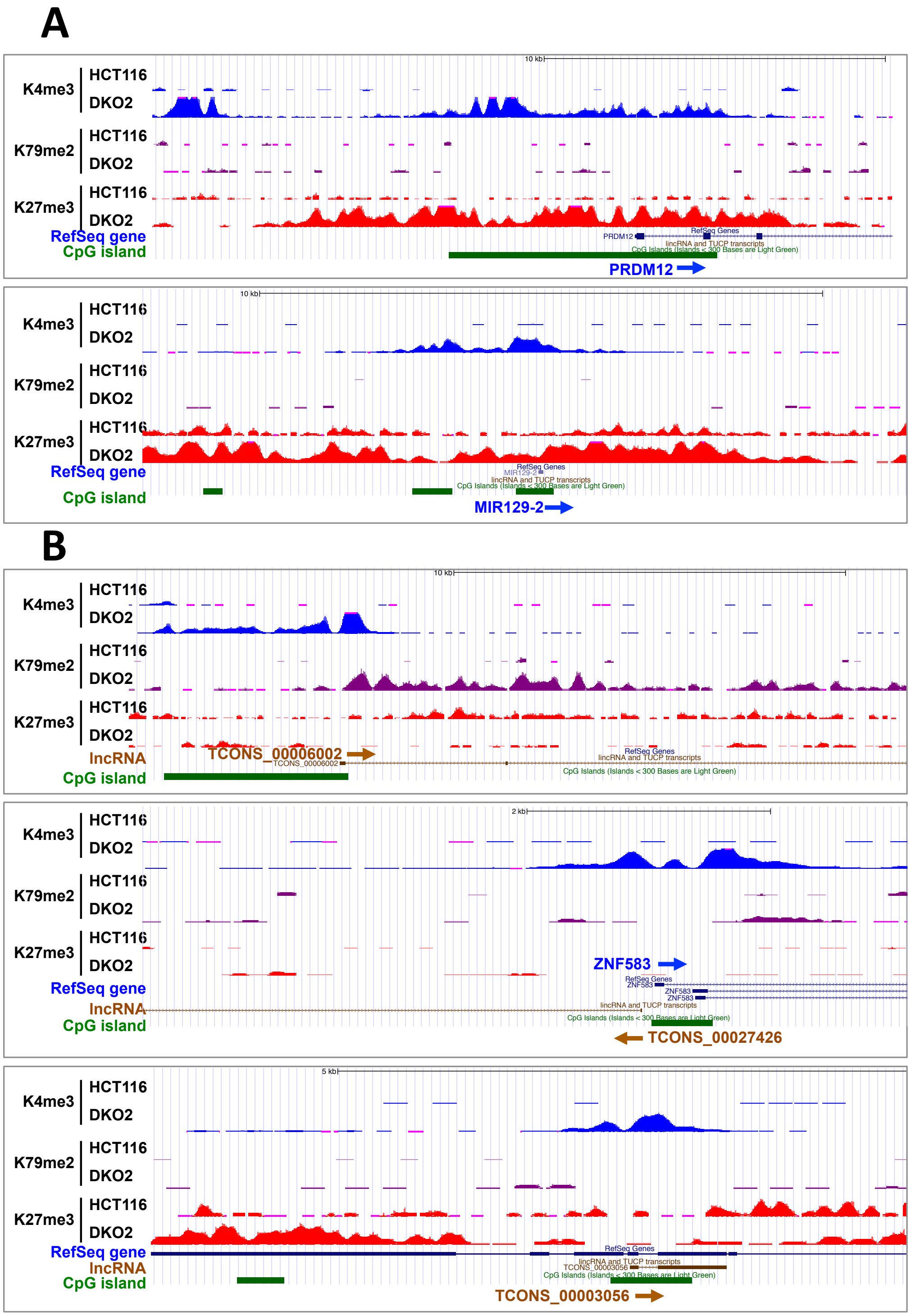

Supplementary Figure S1. Representative ChIP-seq analysis in HCT116 and DKO2 cells. (A) Results for a protein-coding gene (PRDM12) and a miRNA gene (miR-192-2). (B) The results for the lncRNA genes *TCONS_00006002*, *TCONS_00027426* and *TCONS_00003056*.

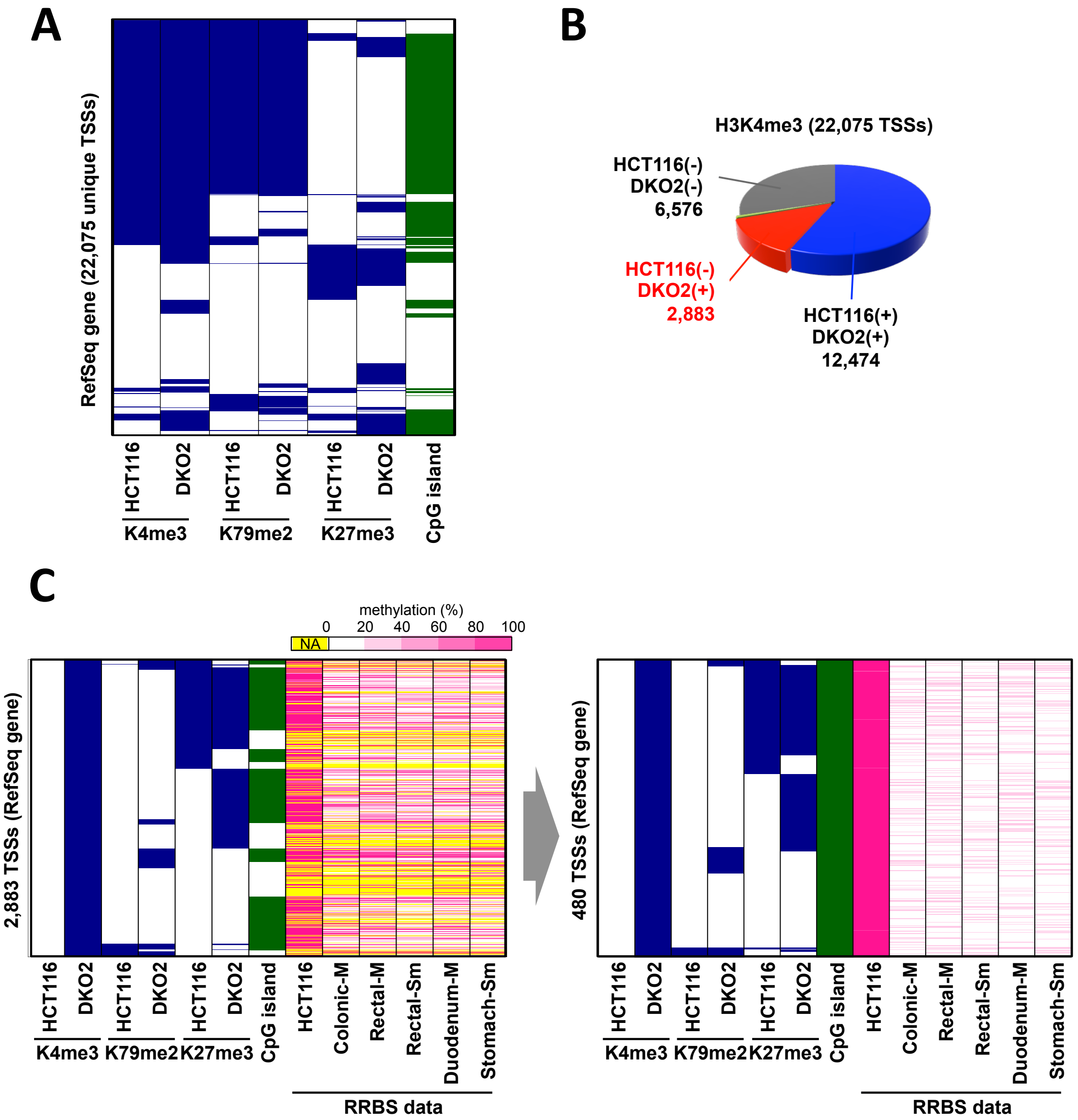

Supplementary Figure S2. Integrative epigenome analysis to screen for epigenetically silenced genes in CRC. The same workflow shown in Figure 1 was applied to RefSeq genes. (A) Heat map showing the presence (blue) or absence (white) of histone modifications (H4K4me3, H3K79me2 and H3K27me3) at the TSS regions of RefSeq genes in HCT116 and DKO2 cells. The presence (green) or absence (white) of a CGI is indicated on the right. (B) Fraction of TSSs with an H3K4me3 mark in HCT116 and DKO2 cells. Shown are the numbers of TSSs with the indicated H3K4me3 status in the two cell lines. (C) Heat maps showing the histone modifications at selected TSSs in CRC cells; also shown is the DNA methylation status obtained from the RRBS data sets in HCT116 and normal gastrointestinal tissues. A set of 2,883 TSSs with increased H3K4me3 in DKO2 cells is shown on the left, and 480 TSSs with cancer-specific CGI methylation are shown on the right. Colonic-M, colonic mucosa; Rectal-M, rectal mucosa; Rectal-Sm, rectal smooth muscle; Duodenum-M, duodenum mucosa; Stomach-Sm, stomach smooth muscle; NA, not available.

A

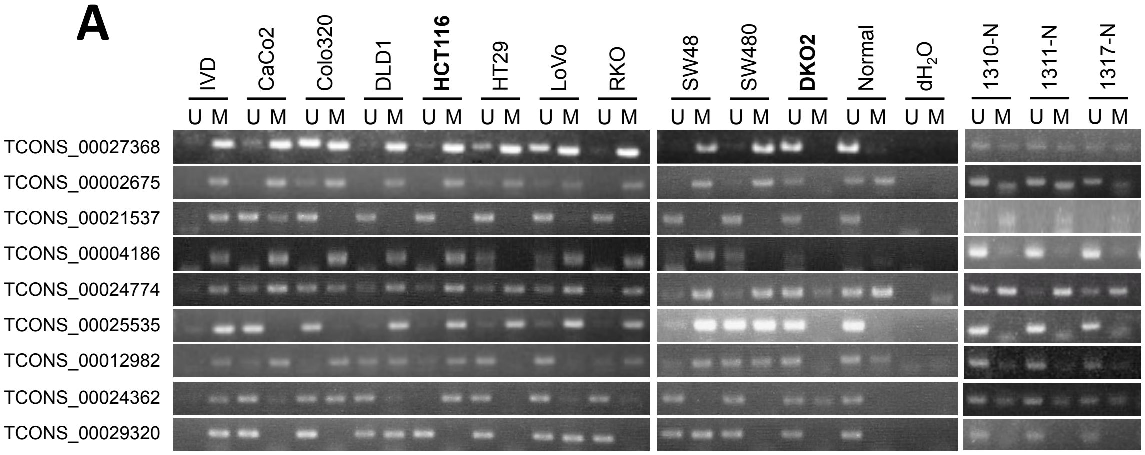

B

Methylation (%)		Sample		
		1310-N	1311-N	1317-N
lncRNA	TCONS_00027118	13.4	13.5	0.0
	TCONS_00027426	3.6	2.3	3.4
	TCONS_00003056	26.6	33.8	42.0
	TCONS_00006002	10.6	12.3	12.7

Supplementary Figure S3. DNA methylation of lncRNA genes in CRC cells. (A) Methylation-specific PCR analysis of lncRNA genes in CRC cell lines, a normal colonic tissue from a healthy individual (24 yo), and normal colonic tissues from CRC patients (1310-N, 74 yo; 1311-N, 75 yo; 1317-N, 79 yo). Bands in the "M" lanes are PCR products obtained with methylation-specific primers. Those in the "U" lanes are products obtained with unmethylation-specific primers. In vitro-methylated DNA (IVD) served as a positive control. (B) Results of bisulfite pyrosequencing of the four selected lncRNA genes in normal colonic tissues from CRC patients.

A

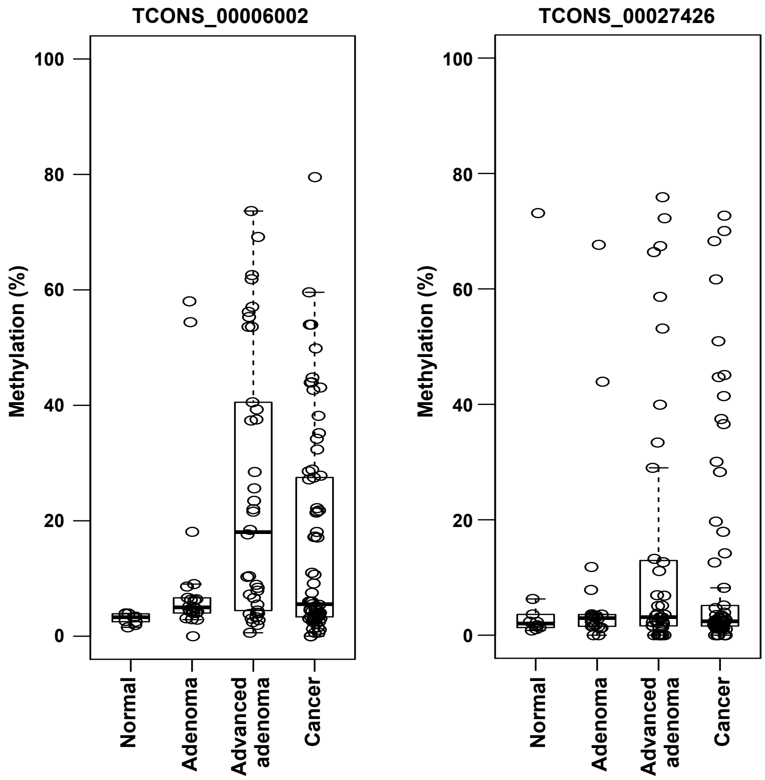

B

TCONS_00006002

	No. of samples	Median of methylation (%)	No. of methylation positive samples (>15%)
Normal	9	3.29	0 (0%)
Adenoma	22	5.00	3 (13.6%)
Advanced adenoma	38	18.0	20 (52.6%)
Cancer	69	5.55	27 (39.1%)

TCONS_00027426

	No. of samples	Median of methylation (%)	No. of methylation positive samples (>15%)
Normal	10	2.02	1 (10.0%)
Adenoma	22	2.97	2 (9.09%)
Advanced adenoma	39	3.13	9 (23.1%)
Cancer	70	2.41	14 (20.0%)

Supplementary Figure S4. Methylation of *TCONS_00006002* and *TCONS_00027426* in clinical samples. (A) Summary of bisulfite pyrosequencing results for *TCONS_00006002* and *TCONS_00027426* in normal colon (n = 46), colorectal adenomas (n= 38), advanced adenomas (n = 40) and primary CRCs (n = 101). Each dot represents a single specimen, and the first, second and third quartiles are shown as box plots. (B) Tables showing the bisulfite pyrosequencing results for *TCONS_00006002* and *TCONS_00027426*.

A

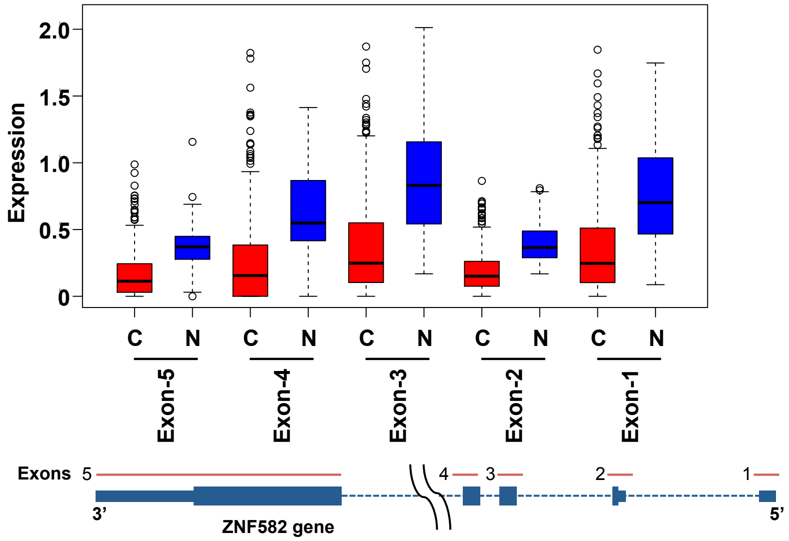

B

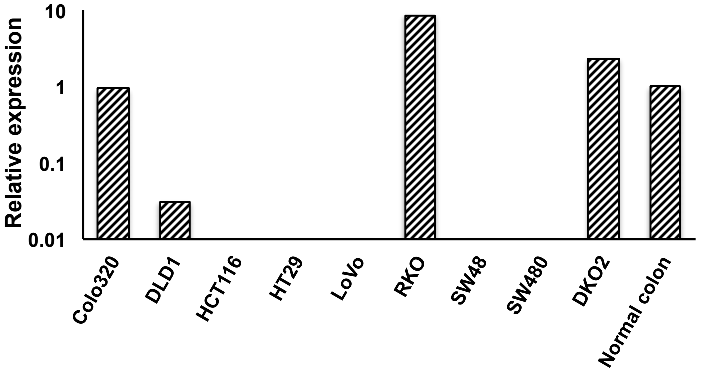

C

Probe	CGI	Genebody	R	P value
cg01772700	no CGI	ZNF582	0.093	1.31E-01
cg24733179	no CGI	ZNF582	-0.224	2.38E-04
cg11740878	CGI	ZNF582	-0.272	6.94E-06
cg09568464	CGI	None	-0.403	8.33E-12
cg02763101	CGI	None	-0.352	3.73E-09
cg22647407	CGI	None	-0.395	2.18E-11
cg08464824	CGI	None	-0.381	1.22E-10
cg13916740	CGI	None	-0.406	5.94E-12
cg24039631	CGI	None	-0.383	1.05E-10
cg20984085	CGI	None	-0.421	7.47E-13
cg25267765	CGI	ZNF582-AS1	-0.438	6.61E-14
cg07135042	CGI	ZNF582-AS1	-0.405	6.60E-12
cg07778983	no CGI	ZNF582-AS1	-0.213	4.61E-04

Supplementary Figure S5. *ZNF582* expression in CRC. (A) Expression levels of the indicated ZNF582 exons in normal colon (n = 50) and primary CRCs (n = 364) analyzed using TCGA data sets. Shown below is the structure of ZNF5821 and the regions analyzed using RNA-seq. (B) Quantitative RT-PCR analysis of ZNF582 expression in CRC cell lines and a normal colonic tissue. (C) Correlations between DNA methylation at the indicated probe sets and expression of ZNF582-AS1 exon 1. The Pearson correlation coefficients and P values are shown.

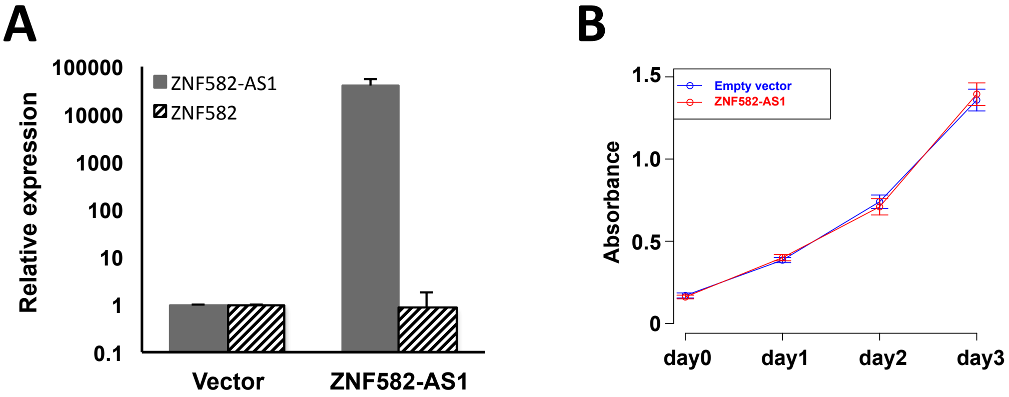

C

GO Term	p value
G-protein coupled receptor signaling pathway	1.55E-08
signaling receptor activity	2.84E-08
transmembrane signaling receptor activity	8.45E-08
receptor activity	9.01E-08
intrinsic component of membrane	2.35E-07
cell periphery	5.32E-07
plasma membrane	5.33E-07
signal transducer activity	7.57E-07
molecular transducer activity	7.57E-07
integral component of membrane	6.49E-07
cell adhesion	1.40E-05
biological adhesion	1.60E-05
G-protein coupled receptor activity	1.78E-05

D

Pathway	p value
GPCR_ligand_binding_WP1825	1.30E-04
Thyroxine_Production_WP1981	0.00372
Fatty_Acid_Omega_Oxidation_WP206	0.00786
Peptide_GPCRs_WP24	0.00940
Folate-Alcohol_and_Cancer_Pathway_WP1589	0.00952

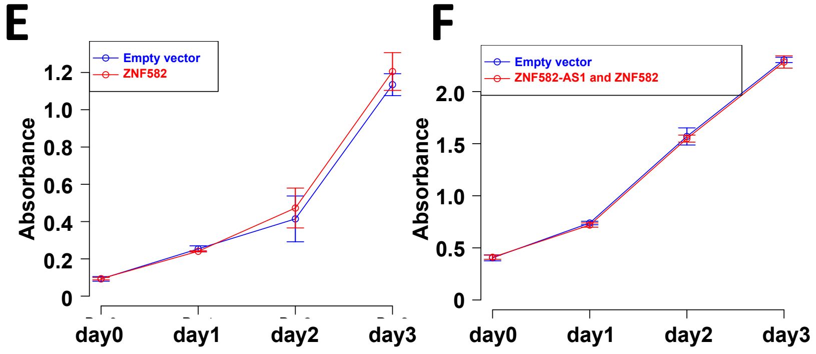

Supplementary Figure S6. Functional analysis of ZNF582-AS1 in CRC cells. (A) Quantitative RT-PCR analysis of ZNF582-AS1 and ZNF582 in CRC cells transfected with a ZNF582-AS1 expression construct or a control vector. (B,E,F) HCT116 cells were transfected with expression constructs encoding ZNF582-AS1 (B), ZNF582 (E) or both (F), or a control vector, after which cell viability was assessed in WST assays at the indicated time points. (C,D) Gene ontology (GO) terms (C) and pathways (D) enriched for downregulated genes by ectopic expression of ZNF582AS1 in HCT116 cells.
